# The effect of autonomous and controlled motivation on self‐control performance and the acute cortisol response

**DOI:** 10.1111/psyp.13915

**Published:** 2021-08-08

**Authors:** Richard P. Steel, Nicolette C. Bishop, Ian M. Taylor

**Affiliations:** ^1^ School of Sport, Exercise and Health Sciences Loughborough University Loughborough UK; ^2^ Department of Psychology School of Social Sciences Nottingham Trent University Nottingham UK

**Keywords:** ego‐depletion, organismic integration theory, self‐determination theory, self‐regulation, stress

## Abstract

Autonomously regulated self‐control typically does not reduce over time as much, compared with self‐control underpinned by controlled motivation. The proposed study tested whether an acute stress response is implicated in this process. Utilizing a framework grounded in self‐determination theory, this study examined whether participants' motivational regulation would influence repeated self‐control performance and acute stress levels, measured by the stress hormone cortisol. A single‐blind randomized experimental design incorporating two motivational conditions (autonomous regulation and controlled regulation) tested these hypotheses. Participants (female = 28; male = 11; *M*_age_ = 22.33) performed three sequential self‐control tasks; a modified Stroop task followed by two “wall sit” postural persistence tasks. Salivary cortisol was measured at baseline and after each of the wall sits. A repeated measures ANCOVA unexpectedly revealed that participants in the controlled regulation condition recorded greater wall sit performance in the first and second wall sits, compared with the autonomous condition. A repeated measures ANCOVA also revealed a significant quadratic interaction for cortisol. Controlled regulation was associated with an increase, and autonomous regulation condition a decrease, in cortisol that subsided at timepoint two. Results imply autonomous motivation facilitates an adaptive stress response. Performance on the self‐control tasks was contrary to expectations, but may reflect short‐term performance benefits of controlled motivation.

## Abstract 2

In the first study to examine the effect of manipulating motivation on the cortisol response and task performance, the results were consistent with previous research as high quality motivation significantly attenuated the cortisol response. However, contrary to expectations, low quality motivation increased task performance. In summary, low quality motivation improved task performance, but was accompanied by an increased physiological cost.

## 1 INTRODUCTION

There are several epistemologically similar models of motivation that suggest the quality of motivation (i.e., the content) is equally, if not more, important than the quantity of motivation (i.e., the magnitude) for goal‐directed behavior and well‐being. For example, the threat of a punishment or promise of a tempting reward would typically be considered as powerful motivators of behavior, especially in the short term. Nonetheless, these motives are poor quality because they will be likely accompanied by psychological costs (Ryan & Deci, 2006), and unlikely to sustain behavior in the long term (Ryan & Deci, 2019). These examples may be viewed as exceptional cases; however, many other types of motivation can be considered a double‐edged sword by facilitating behavior on the one hand, but at the expense of well‐being on the other. Researchers have recently begun to adopt this perspective to examine the underlying physiological impact of different qualities of motivation, providing the opportunity for greater understanding of the interplay between psychological and physical health. This study builds on this movement by examining the concurrent effect of different qualities of motivation on performance during physical persistence tasks and salivary cortisol secretion, a biomarker of acute stress.

One of the leading theories that seeks to describe varying quality of motivational regulation is self‐determination theory, particularly the subtheory, organismic integration theory (OIT; Ryan & Deci, 2017). The theory postulates that humans are growth‐oriented and will, under appropriate conditions, naturally internalize externally sanctioned behavior to become personally endorsed, valued, and self‐regulated (Ryan et al., 1985). The degree to which this process is completed or forestalled generates varying qualities of behavioral regulation. Behavior that is successfully internalized is regulated autonomously, with a sense of volition and choice. Autonomous motivation can encompass two broad types of regulation. Identified regulation refers to behavior that is personally endorsed as being valuable, for example, consuming nutritious food choices because it is important to eat healthily. Integrated regulation reflects behavior that is fully endorsed and integrated with all aspects of person's wider identity (Ryan & Deci, 2019). For example, when healthy eating is an expression of an individual's true sense of self.

In contrast, partially internalized behavior or behavior that remains externally regulated is controlling in nature (Ryan & Deci, 2017). External regulation is the most controlled form of regulation (hence, the least internalized form of regulation) and refers to engagement in an activity that is contingent on external rewards or to avoid punishment (Ryan & Connell, 1989). An example is a person who undertakes a task only to receive payment or avoid punitive measures. Introjected regulation, on the other hand, refers to behavior that has been partially internalized, yet is still controlling because the pressurizing contingencies are administered by the individual, rather than from external sources. For example, participating in an exercise act primarily to avoid guilt or to obtain self‐worth (Deci & Ryan, 2000).

There exists a body of literature that supports the beneficial outcomes of high‐quality autonomous motivation, compared with low‐quality controlled motivation, in many life contexts such as education (see Ryan & Deci, 2020), sport (see Taylor, 2015), health behavior change (Ntoumanis et al., 2021), and relationships (see Legault & Amiot, 2014). A feature of autonomous motivation is that it is less demanding on self‐regulatory resources, compared with controlled motivation. For example, autonomously motivated goals are also more effortlessly activated and pursued (Carver & Scheier, 2018; Werner et al., 2016). Autonomous motivation also leads to lower temptation away from the pursuit of personal goals; therefore, less self‐control is required to resist temptation (Milyavskaya et al., 2015; Taylor et al., 2020). Self‐control typically declines over repeated use, a process termed “ego‐depletion” (Baumeister et al., 2018). The depletion of self‐control is typically measured using the sequential task paradigm. In this procedure, the experimental group performs two different tasks requiring the exertion of self‐control. Meanwhile, the control group performs an identical second task; however the first task, which was conceptually similar to the experimental group, does not require self‐control exertion. Performance between groups on the second task is then compared, with the difference in performance assumed to represent the additional exertion of self‐control on the first task. Due to the lower cognitive demands, however, autonomous motivation can moderate this ego‐depletion effect (Muraven, 2008). Ego‐depletion is reduced when participants are given contextual support for their autonomous regulation. Examples of contextual support include provision of appropriate rationales for the importance of the task, being led to believe that performance would improve the longer they practiced (Muraven & Slessareva, 2003), free choice (Moller et al., 2006), or receiving autonomy supportive instructions for the task (Muraven et al., 2008).

In addition to the reduced cognitive demand, autonomous motivation may also lead to an, as yet untested, second response to repeated use of self‐control. It has been suggested that stress depletes self‐control resources, but results are inconclusive (Baumeister & Vohs, 2016). In addition to reducing ego‐depletion, autonomous motivation also attenuates the stress response when compared with controlled motivation (Reeve & Tseng, 2011). It is therefore plausible that autonomously motivated individuals benefit from a lower stress response, and therefore greater self‐control resources are subsequently available to cope with future demands. In this study, the focus is on cortisol; a key hormone activated during the stress response which has important implications for optimal human functioning. In response to a stressful situation, the hypothalamic pituitary adrenal axis stimulates the release of cortisol via the adrenal cortex. Cortisol subsequently stimulates lipolysis, gluconeogenesis, and catabolism of proteins into amino acids. In turn, this helps to repair damaged tissue and produce adenosine triphosphate, the body's key currency used in energy transfer. In sum, these processes mobilize appropriate resources to meet the demands of the stressor (McEwen, 1998). However, although cortisol is adaptive in helping the body to deal with short‐term stress, prolonged exposure to stress can lead to psychological dysregulation (e.g., Burke et al., 2005), and impair other physiological process such as immune functioning (Dhabhar, 2014). Thus, examination of cortisol represents a valid and reliable method of investigating how motivational processes are implicated in downstream physiological processes that are associated with adaptive human functioning but may also have health implications. Research has demonstrated the potential link between autonomous motivation and cortisol responses. For example, an autonomy supportive learning environment attenuated the cortisol response, whereas a learning environment facilitating controlled motivation amplified the cortisol response, relative to a neutral condition (Reeve & Tseng, 2011). However, the effects of motivation regulation on the cortisol response during self‐control efforts are unknown. Identifying this process may provide a second explanation of why autonomous regulation mitigates ego‐depletion, beyond more efficient use of cognitive resources; in other words, it attenuates the stress response.

To summarize, several studies have demonstrated a link between higher quality motivation and less self‐control depletion (e.g., Moller et al., 2006). This effect is currently explained by a more efficient use of self‐regulatory resources (e.g., Milyavskaya et al., 2015). However, another potential explanation is that autonomous motivation may reduce the stress response, thus leaving more resources available to meet the demands of tasks requiring self‐control exertion. Hence, the goal of this study was to examine how autonomous and controlled motivation influence (a) repeated self‐control performance (i.e., ego‐depletion) and (b) the cortisol response during repeated self‐control exertion. As has been observed in previous work (e.g., Moller et al., 2006), it was hypothesized that there will be no performance difference on an initial self‐control task under autonomous and controlled motivation conditions (hypothesis one). However, during a second self‐control task, participants in the autonomous condition will perform better than those in the controlled regulation condition. In other words, participants in the autonomous condition will experience less ego‐depletion than participants in the controlled regulation condition, thus leaving more resources available for further self‐control tasks (e.g., Moller et al., 2006; hypothesis two). Furthermore, it has been suggested that motivational effects on ego‐depletion may dissipate over time (Graham et al., 2014). Hypothesis three, therefore, examined if any ego‐depletion effects observed in the second self‐control task would persist during a third self‐control task. Finally, based on previous research (Reeve & Tseng, 2011), it was predicted that autonomously motivated participants would experience a decreased cortisol response during the self‐control tasks, compared with the controlled regulation participants (hypothesis four).

## 2 METHOD

### 2.1 Participants

With an experimental design of a two (autonomous vs. controlled motivation) × three (cortisol) repeated measures ANOVA, it was determined that a minimum sample size of 30 participants would likely be sufficient (*α* = .05, *β* = .80, *f* = .80; G*power; Faul et al., 2007). The effect size (*f*) was calculated from a similar study manipulating autonomous and controlled motivation as the independent variable and measuring cortisol as the repeated measures outcome (*η* ^2^ = .33; Reeve & Tseng, 2011). Further justifying the use of a large effect size, a recent systematic review revealed the manipulation of high and low quality motivation with the intention of detecting repeated‐measures differences in cortisol consistently detected large effect sizes (Steel et al., 2021). Data were subsequently collected from 41 participants; however, two participants were removed from the analysis. One participant did not choose the required option in the controlled condition. The other participant was removed as they appeared behaviorally disengaged during the Stroop task, and on further investigation recorded 138 “no responses,” which was a significant outlier across the sample (*M* = 46.30; *SD* = 28.80). Analyses were subsequently performed on 39 participants (female = 28; male = 11; *M*_age_ = 22.33). Given the known sample size and correlation between measures, we conducted a sensitivity power analysis to determine the minimum effect size detectible for all hypotheses. Effect size sensitivity analysis is recommended as the most useful and honest tool in determining power, particularly postreplication crisis (Cohen, 1988; Giner‐Sorolla et al., 2019). The calculation for hypothesis four was determined by the sample size of 39, assigned to one of the two groups, with 80% power at an alpha = .05 and the median correlation between repeated measures cortisol of .82. For the difference between autonomous and controlled groups, the minimal detectable effect was Cohen's *f* = .43, or *η*_p_^2^ = .16. The same calculation was undertaken to determine the sensitivity power analysis of the wall sit (hypotheses one to three). The number of repeated measures was reduced to two and correlation between measures increased to .65 (see Table 1). The minimal detectable effect between groups was Cohen's *f* = .42, or *η*_p_^2^ = .15. For recruitment, participants were invited to take part in a study measuring “The effect of cognitive and physical performance on hormones,” and qualifying undergraduate students were eligible to receive course credit for participating.

**TABLE 1:** Descriptive statistics for the variables between autonomy support and controlled regulation conditions

	Autonomy (*n* = 19)	Controlled (*n* = 20)																
	*M(SD)*	*M(SD)*	*t* =	*p* =	1	2	3	4	5	6	7	8	9	10	11	12	13	14
1. IAF	3.30 (.43)	3.52 (.56)	1.35	.19														
2. IMI	3.33 (.39)	3.31 (.50)	.13	.90	−.03													
3. BMIS	3.05 (.45)	2.99 (.37)	.42	.68	−.08	.26*												
4. Mental Borg 1	5.68 (1.57)	5.58 (2.12)	.18	.86	.05	.01	−.23											
5. Physical Borg 1	5.42 (2.89)	6.10 (1.92)	.87	.39	.00	.14	.05	.40*										
6. Mental Borg 2	2.50 (1.30)	3.08 (2.15)	1.00	.32	.16	−07	−.30*	.40*	.18									
7. Physical Borg 2	5.97 (2.86)	6.75 (1.59)	1.06	.30	−.94	.01	.05	.39*	.85*	.31*								
Modified Stroop																		
8. Correct response	285.00 (30.29)	282.25 (33.19)	.27	.79	−.19	.02	.33*	−.37*	−.13	−.15	−.16							
9. Incorrect response	29.58 (20.09)	31.80 (24.13)	.31	.76	.10	−.29*	−.21	.43*	.32*	.36*	.26	−.47*						
10. No response	45.42 (22.46)	45.95 (34.14)	.06	.96	.14	.20	−.21	.08	−.10	−.11	−.20	−.74*	−.25					
Congruent Stroop																		
11. Correct response	342 (9.68)	341.20 (10.73)	.24	.81	−.13	.14	.19	−.37*	−.11	−.49*	−.18	.58*	−.50*	−.25				
12. Incorrect response	14.56 (9.18)	13.80 (8.76)	.26	.80	.05	−.24	−.04	.29*	.24	.40*	.29*	−.31*	.61*	−.13	−.85*			
13. No response	3.44 (4.00)	5.00 (6.31)	.90	.38	.17	.12	−.29*	.22	−.20	.27*	−.14	−.60*	−.07	.69*	−.49*	−.05		
14. Wall sit 1^a^	124.32 (53.73)	157.80 (62.18)	1.80	.08	−.06	.08	.29*	−.20	.33*	−.26	.29*	.19	−.01	−.20	.06	.09	−.27*	
15. Wall sit 2^a^	97.00 (41.58)	125.75 (51.57)	1.91	.06	.01	.09	.22	−.16	.20	−.20	.17	−.39	−.15	−.15	−.03	.19	−.25	.65*

Abbreviations: BMIS, brief mood introspection scale; IAF, index of autonomous functioning; IMI, intrinsic motivation inventory.

^a^ Measurement in seconds.

*p < .05.

### 2.2 Procedure

**FIGURE 1:**
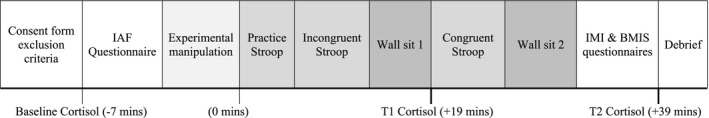
Graphical illustration of the experimental timeline

Data collection was undertaken at either or 11:00 a.m. or midday to control for the cortisol diurnal profile. A summary of the study procedure can be seen in Figure 1. Prior to partaking in the experiment, participants were asked to refrain from or document activities that may affect performance or cortisol reactivity. Participants were instructed to abstain from brushing their teeth for 30 min, consuming food or drink other than water for one hour, and to not consume a major meal at least two hours previously. They were also asked to document any alcohol or nicotine consumption and indicate whether they had undertaken any physical activity in the 12 hr prior to the study. Participants were also asked to provide details of any prescription medication that they were taking, and their awakening time that day. Participants then provided a baseline sample of saliva (to measure cortisol) and self‐reported trait autonomy (secondary variable, see Measures section).

Participants were then assigned into either autonomous or controlled regulation experimental conditions. Odd‐numbered participants were assigned to the autonomous condition, with even numbered participants assigned to the control condition. In a procedure adopted from Legault and Inzlicht (2013), participants were offered a choice of a forthcoming task. The options were (a) The Mental Distraction Game; (b) A Game of Accuracy; (c) Ignore Your Impulses, and (d) Cognitive Response Latency Test. Unbeknown to the participants, these were all different names for a modified Stroop task (Wallace & Baumeister, 2002) they would all perform. In both conditions, participants were presented with the four options printed on a sheet of A4 paper. Participants in the autonomous condition were offered a free choice between the options. It was emphasized that they would receive feedback to indicate how successful they were to enhance the personal relevance (i.e., autonomy) of the task.

Participants in the controlled regulation condition were presented with the same options offered in the autonomous condition. However, upon presentation of the four options, the experimenter verbally explained that most participants had chosen options “a,” “b,” or “c,” and that it would help the researcher to balance the experimental conditions if they chose option “d,” the Cognitive Response Latency Test, which was ostensibly the least desirable task. In the autonomy‐supportive condition only two participants freely chose this option. In contrast, all but one of the participants was verbally coerced into completing option “d”. This difference suggests that participants in the controlled condition were manipulated into a choice they would most likely not have chosen if pressure were absent. This process was designed to induce a feeling of guilt and coercion when selecting the task (i.e., controlled regulation). Aside from this experimental manipulation, all other aspects of the autonomous and controlled conditions were identical.

Participants then engaged in a series of the three self‐control tasks. The first self‐control task was a modified Stroop task written using Superlab (v4.5; Cedrus Corporation, San Pedro, CA), with responses made on a Cedrus RB‐530 response pad. The modified Stroop task has been employed in numerous self‐control studies (Hagger et al., 2010) and can elicit a physiological stress response (Renaud & Blondin, 1997). Nine blocks of forty color words written in either red, green, blue, and yellow were displayed on a computer screen for a total of 800 ms, with a 500 ms pause between words where a control “+” was displayed in the center of the screen in black.

Participants were required to respond to the color the word was written in and to use self‐control to override the impulse to respond to the written name of the color (e.g., for the word “GREEN” displayed in the color yellow, the correct answer would be “yellow”). The exception to this rule was when the ink color was red; participants were then required to respond with the semantic meaning of the word, not the color of the text (i.e., the word “GREEN” in red ink, the correct response would be “green”). Additional self‐control is required to override the general rule to identify the color of the text, and instead identify the semantic meaning of words written in red ink. Participants were allowed a practice consisting of 40 words with 1,500 ms to input a response, with a 500 ms pause between words. Participants were allowed as many trials as they wanted to ensure they understood the task and given the opportunity to ask questions to clarify any aspects. When they were ready to proceed, participants began the experimental task, which ran for approximately 10 min. On completion of the modified Stroop, participants indicated the mental exertion the task required (secondary variable).

Reductions in self‐control over time (i.e., ego‐depletion) are typically examined using the sequential task paradigm in which participants complete different tasks requiring self‐control. Hence, the second self‐control task was a wall‐sit postural endurance task, which requires self‐control exertion to override a desire to stop to relieve the discomfort (Boat & Taylor, 2017). The procedure required the participant to place their back and shoulders against a wall within the laboratory and assume a sitting position with their knees bent at 90° and their thighs parallel to the floor. Once they had assumed the position, the experimenter started a stopwatch and they were required to hold this position for as long as they were able. Failure was deemed to occur when the participant's form deviated from the original position, and they were unable to correct within two seconds when verbally prompted by the experimenter. Once failure had occurred, the total time elapsed was recorded to the nearest second. Upon completion, participants indicated how much physical exertion the wall sit had required (secondary variable) and provided a second saliva sample.

Next, a congruent Stroop was administered to standardize the activity of participants between the second and third saliva samples. This task was administered to ensure that participants would not engage in other activities that might affect their stress levels (e.g., check their mobile phone). The congruent version of the Stroop included colors and words that were congruent throughout the task (i.e., the word BLUE in blue ink). The congruent Stroop does not require self‐control exertion (Hagger et al., 2010), and when presented after the modified Stroop is not stressful (Renaud & Blondin, 1997). Upon completion, participants indicated how much mental exertion the task required.

Participants were then asked to perform a second identical wall sit task as the third measurement of self‐control. Upon completion, participants indicated how much physical exertion the task required. For the final part of the experiment, participants completed measures of intrinsic motivation, mood (secondary variables), and a third saliva sample. Participants were then debriefed and thanked for their participation.

### 2.3 Measures

#### 2.3.1 Self‐control

Self‐control performance was measured by correct answers, number of errors, and response times during the incongruent Stroop task (Task one) and performance in seconds on the wall‐sit tasks (Task two and three). The modified Stroop is a valid and reliable measure of ego‐depletion, yielding moderate‐to‐large effect sizes when employed as the depleting (*d* = .40) and dependent (*d* = .61) task (Hagger et al., 2010; however also see Carter & McCullough, 2014 for discussion of ego deletion effect sizes). The wall sit has also been successfully employed as the depended task in self‐control studies (e.g., Boat et al., 2018).

#### 2.3.2 Cortisol

Following the protocols outlined by the immunoassay kit manufacturer (Salimetrics, State College, PA, USA), saliva was collected via the passive drool method. Participants were seated, asked to empty their mouth, and allow saliva to pool in the well of the mouth without stimulation via facial movement. With their head tilted slightly forward, they were instructed to drool into a 15 ml cryovial approximately every 60 s. A target was set of 5 ml of saliva collected over approximately three minutes. As the saliva was collected unstimulated, it took some participants slightly longer to provide an adequate sample. Saliva samples were taken at three timepoints, hereafter referred to as baseline, timepoint one (+19 min), and timepoint two (+39 min). Upon completion of the experiment, samples were immediately stored at −80℃ until assay.

Analysis of the cortisol samples was undertaken by the lead author using a commercially available salivary cortisol enzyme immunoassay kit (Salimetrics, State College, PA, USA). On the day of assay, the samples were thawed at room temperature (~22℃) for a minimum of 1.5 hr. Upon thawing, 1,000 µl of saliva was centrifuged at 10,000 rpm for two minutes. The samples were analyzed in duplicate in accordance with the manufacturer's instructions. The immunoassay measured cortisol in 25 µl wells, with a sensitivity of <0.007 µg/dl. Intra‐assay and interassay precision coefficient of variation were 4.6% and 6%, respectively. Spike recovery across eight samples averaged 104.9%, dilution recovery averaged 105.3% (four samples) and linearity of assay averaged 101.3% (nine samples).

#### 2.3.3 Secondary variables

##### Trait autonomy

The Index of Autonomous Functioning (IAF; Weinstein et al., 2012) was used to rule out initial group differences in trait autonomy. The 15‐item scale consists of three subscales; self‐congruence (five items: e.g., “I strongly identify with the things that I do”); susceptibility to control (five items: e.g., “I do things in order to avoid feeling badly about myself”); and interest taking (five items: e.g., “I like to investigate my feelings”). Participants were asked to rate their general experiences toward each of the statements on a 5‐point Likert scale ranging from 1 *(Not at all true*) to 5 (*Completely true*) and all items were summed to provide a total trait autonomy score. The scale has demonstrated good internal consistency and reliability across a range of studies (*α* = .81, ICC = .86; Weinstein et al., 2012).

##### Mental and physical exertion

The Borg single‐item CR‐10 scale (Borg, 1998) was used to measure perceived mental and physical exertion on the Stroop tasks and wall sit, respectively. The scale was administered to assess if there was a difference in perceived exertion between experimental conditions, and between the congruent active control and incongruent Stroop. The scale uses a single 10‐point scale, with higher scores indicating more perceived physical exertion (0 = *extremely weak*; 10 = *absolute maximum*). The Borg scale has been successfully used in similar studies as a measure of perceived exertion (e.g., McEwan et al., 2013).

##### Intrinsic motivation

The Intrinsic Motivation Inventory (IMI; McAuley et al., 1989) was administered to assess if participants reported different levels of intrinsic motivation after the experiment had been completed. The questionnaire consists of three subscales rated on a 5‐point Likert scale ranging from 1 (*Not at all true*) to 5 (*Completely true*): interest/enjoyment (eight items: e.g., “This activity was fun to do”), value/usefulness (nine items: e.g., “I think this is an important activity”), and perceived choice (eight items: e.g., “I felt that I had to do this activity”). The three subscales were summed to provide a total score for intrinsic motivation. The scale has shown good internal consistency in similar studies measuring intrinsic motivation toward computer tasks (*α* = .93; Moller et al., 2006).

##### Mood

The Brief Mood Introspection Scale (BMIS; Mayer & Gaschke, 1988) assessed mood to ensure any experimental effects were not attributable to differences mood at the end of the experiment. Participants rated 16 items (e.g., “lively,” “drowsy”) describing how well each item described their present mood, using a 4‐point Likert scale ranging from 1 (*definitely do not feel*) to 4 (*definitely feel*). Items describing negative affect were reverse scored when calculating the total score. The scale has good internal consistency (*α* = .76–.83; Mayer & Gaschke, 1988).

### 2.4 Data analysis

To test the first hypothesis, a series of independent sample *t* tests were conducted to examine for group differences in Stroop performance (correct, incorrect, and no responses). Before testing the second, third, and fourth hypotheses, a multiple linear regression was conducted to test for potential covariates of wall sit performance (hypotheses two and three) or cortisol reactivity (hypothesis four), with significant predictors subsequently included as covariates in ANCOVA. Gender, age, and physical activity undertaken in the previous 12 hr were analyzed as covariates of self‐control performance on the first wall sit. Potential covariates for cortisol analysis included consumption of food or drink other than water for one hour; to not eat a major meal at least two hours; any nicotine or alcohol consumed in the previous 12 hr; any physical activity undertaken in the previous 12 hr; a list of any medication; and hours awake at baseline cortisol. The second and third hypotheses were subsequently tested using a repeated measures ANCOVA, with experimental condition as the independent variable, the two wall sit tasks as the repeated measures dependent variable and any significant covariates. The fourth hypothesis was also tested using a repeated measures ANCOVA, with experimental condition as the independent variable, and cortisol as the repeated measures dependent variable together with any significant covariates.

## 3 RESULTS

### 3.1 Preliminary analysis

All data were normally distributed and had homogenous variances across conditions, aside from a positively skewed distribution of the cortisol samples. Following established guidelines (Smyth et al., 2013) four participants were removed from the cortisol analysis due to awakening less than 60 min before the experiment (one case), or their baseline cortisol being greater than three standard deviations above the mean (three cases). Despite the removal of these outliers Shapiro‐Wilk tests indicated that cortisol at baseline (*W* (17) = .82, *p* = .004), timepoint one (*W* (18) = .76, *p* < .001) and timepoint two (*W* (18) = .87, *p* = .02) were not normally distributed. Logarithmic (Log‐10) transformation of the data was conducted, which normalized the data at baseline (*W* (18) = .93, *p* = .18), timepoint one (*W* (17) = .95, *p* = .50) and timepoint two (*W* (17) = .95, *p* = .51). Further data checks revealed Levine's test of homogeneity of variance was maintained (*F* (1,33) = .03, *p* = .86), and there was equal covariance across groups (Box's *M* = 6.24, *p* = .47).

Means, standard deviations, differences between experimental conditions and correlations for study variables are presented in Table 1. An independent sample *t* test demonstrated that there were no significant differences between conditions in dispositional autonomy, mood, intrinsic motivation, or mental and physical exertion. These findings suggest that there were no pre‐existing group differences in dispositional autonomy; the self‐control tasks elicited similar exertion across groups; and the experiment did not lead to differences in mood or intrinsic motivation at the end of the trial. Finally, participants reported higher mental exertion following the modified Stroop compared with the congruent Stroop task (*F* (1,37) = 79.18, *p* < .001, *η*_p_^2^ = .68). This validates the relatively greater cognitive effort required by the modified Stroop.

### 3.2 Motivational differences in self‐control performance

Hypothesis one examined whether there were any differences in Stroop performance between the autonomy‐supportive and controlled motivation conditions. As expected, an independent sample *t* test revealed that there were no significant differences in correct answers, incorrect answers, or no responses on the modified Stroop (see Table 1). The second and third hypotheses explored whether there were group differences in performance on the dependent measures of ego‐depletion, the first and second wall sits. Prior to the main analysis, gender, age, and physical activity undertaken in the previous 12 hr were explored as predictors of self‐control performance on the first wall sit, A multiple linear regression with age, gender, and physical activity as predictors of wall sit performance revealed physical activity (*β* = .34; *p* = .04) but not age (*β* = .01; *p* = .93) or gender (*β* = .−.17; *p* = .30) predicted wall‐sit time, therefore, this variable was included as a covariate of self‐control performance.

Data were analyzed using a repeated measures analysis of covariance (ANCOVA), with experimental manipulation (autonomy‐support vs. controlled regulation) as the between‐participants factor, wall sit performance as the repeated measures factor, and physical activity as the covariate. A main effect of experimental condition was significant (*F* (1,36) = 4.40, *p* = .04, *η*_p_^2^ = .11). However, this was in the opposite direction to hypothesis two; participants in the autonomous condition recorded shorter wall sit times compared with the controlled condition. Investigation of the effect of time revealed that performance did not significantly decline over time (*F* (1,36) = 2.91, *p* = .10, *η*_p_^2^ = .08). Finally, the time ×condition interaction was not significant (*F* (1,36) = .14, *p* = .71, *η*_p_^2^ = .01), suggesting the difference in wall‐sit performance did not dissipate over time (Hypothesis three).

### 3.3 Motivational differences in cortisol responses

Prior to conducting the main analysis, potential covariates of cortisol were checked that have the potential to affect data. These included abstinence from smoking or brushing teeth for 30 min; no food or drink other than water for one hour; to not eat a major meal at least two hours; any nicotine or alcohol consumed in the previous 12 hr; any physical activity undertaken in the previous 12 hr; a list of any medication; and hours awake at baseline cortisol. The potential for influence on the cortisol response was checked by regressing the cortisol taken at timepoint one on baseline cortisol and the covariates. Of the potential covariates, only number of hours awake was significant (*β* = −.18; *p* = .04), therefore, this was included this as a covariate in the main model to control for diurnal variation.

**FIGURE 2:**
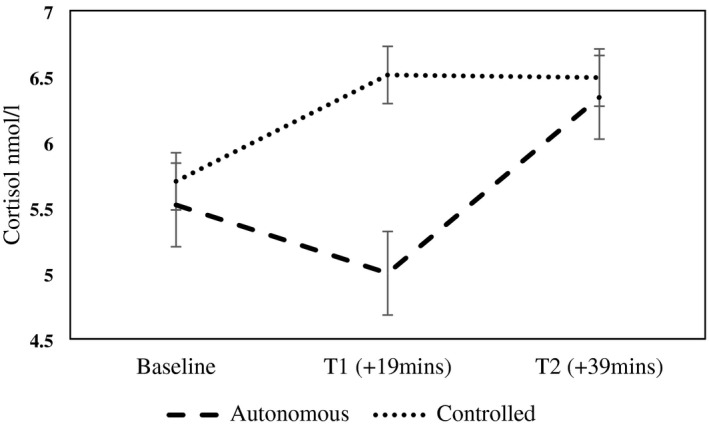
Change in the repeated measures of cortisol between conditions with standard error bars. T1 = timepoint one; T2 = timepoint two. Timepoints are measured in relation to the experimental manipulation. Simple effects *p* > .05 at all timepoints

Due to a violation of sphericity (*χ* ^2^ (2) = 13.77, *p* = .001), the Huynh‐Feldt correction was reported for the main effects. A repeated measures ANCOVA, with experimental condition as the independent variable, cortisol as the repeated measures factor, and waking to baseline saliva time as the covariate showed a nonsignificant within‐person effect (*F* (1.62, 51.91) = 1.70, *p* = .20, *η*_p_^2^ = .05). Furthermore, there was a nonsignificant between‐person experimental effect (*F* (1, 32) = .17, *p* = .68, *η*_p_^2^ = .01). However, there was a significant quadratic time ×condition interaction for the experimental condition on participants' cortisol levels (*F* (1, 32) = 5.40, *p* = .03, *η*_p_^2^ = .14). As can be seen in Figure 2, baseline cortisol values were very slightly higher in the controlled condition than the autonomous condition. This difference was exaggerated at timepoint one, but then returned to a very small difference at timepoint two. Including the outliers discarded during the data check did not meaningfully affect the quadratic effect (*F* (1, 34) = 4.18, *p* = .05, *η*_p_^2^ = .11). The results therefore support experimental hypothesis four.

## 4 DISCUSSION

The present research examined distinct qualities of motivation, namely autonomous and controlled motivation, and their subsequent effect on the cortisol response to a stressful situation and self‐control performance. Overall, poor quality motivation was a double‐edged sword. When motivated to complete a self‐control task through mild coercion and obligation, participants performed better on subsequent physical persistence tasks, but also exhibited a greater stress response.

Despite the results running contrary to hypotheses two and three, there are circumstances in which motivation undertaken for more controlled reasons can still motivate people to achieve positive outcomes in the short‐term, even when compared with more autonomous motivation (Gagné & Deci, 2005). In the most recent meta‐analysis to explore this proposition (Cerasoli et al., 2014), autonomous motivation was associated with higher quality performance (e.g., creativity, assembly quality, writing a research proposal). On the other hand, more controlled forms of motivation were associated with better performance on less complex tasks. For example, tasks that are lower in complexity, require less cognitive investment and where performance can be measured using discrete units of output (Cerasoli et al., 2014; Gilliland & Landis, 1992). Therefore, the characteristics of a wall sit correspond the criteria for less complex tasks. Hence, controlled motivation may have had a more potent effect on the straightforward physical task. Indeed, the original self‐determination theory investigation showed positive performance effects of financial rewards (a form of controlled regulation) in the short term, with deleterious effects only occurring when the reward was removed (Deci, 1971). Our results imply that this short‐term positive effect of controlled regulation may also occur in situations where repeated self‐control is required.

Although controlled motivation may energize short‐term behavior, it is often accompanied by psychological costs (e.g., Ng et al., 2012). Our fourth hypothesis extended this idea to physiological phenomena by predicting that autonomous participants would exhibit a reduced acute cortisol response, compared with the controlled regulation condition. The results supported this proposal. When participants were obliged to choose an ostensibly less favorable self‐control task, they subsequently displayed an increase in cortisol. This extends a growing body of work demonstrating that higher quality motivation is adaptive in reducing the cortisol response in evaluative situations (Steel et al., 2021). In this study, a single “dose” of motivational manipulation was used, and the experiment was identical between conditions in all other aspects. This methodological design contrasts with the previous studies where the motivational salience was consistently reinforced throughout the task (e.g., Reeve & Tseng, 2011). The subtlety of the motivational component manipulation may explain why the effect size was not as large as previous work and why cortisol levels at timepoint two returned to levels that were not statistically different between conditions. As the motivational manipulation was not reinforced throughout the task, it is likely that the motivational salience dissipated by the time the final measure of saliva was taken, 39 min after the experimental manipulation. Indeed, no differences in motivation were observed between conditions at this point in the experiment. It should also be noted that cortisol levels are expected to return to prestressor levels from around 40 min poststressor (Dickerson & Kemeny, 2004).

It is also important that this study was the first to test the effect of high‐quality motivation on cortisol response in individuals participating solo. Previous studies (e.g., Hogue et al., 2017; Reeve & Tseng, 2011; Yeager et al., 2016) conducted their experimental manipulation concurrently in large groups of participants. This has the potential for greater social‐evaluative threat, which has an additive effect on the cortisol response (Dickerson & Kemeny, 2004). By testing participants in isolation, the findings imply that high‐quality motivation modifies the cortisol response in situations where social‐evaluative threat is diminished, albeit the presence of the experimenter meant that social‐evaluative threat would not have been eliminated.

It is also possible that the increased cortisol response in the controlled regulation condition attributed to the unexpectedly better performance on the wall sit tasks. There is little evidence pertaining to the potential interaction between motivational processes, physiological responses, and task performance. However, an evolutionary conceptualization of self‐control offers the suggestion that resources may be allocated, rather than limited, partly via physiological processes (Beedie & Lane, 2012). Cortisol is responsible for mobilizing energy resources to respond to a potential threat (McEwen et al., 2015), therefore, the stress response may have led to allocation of resources to cope with the physical demands of the wall‐sit task. Moderate increases in stress levels can facilitate increased physical performance levels, particularly in disciplines that require strength and gross physical effort (e.g., Crewther et al., 2011). How this positive effect of stress on the physical characteristics of the wall‐sit self‐control task coincides with complex effects of stress on the cognitive demands (McEwen & Sapolsky, 1995; Muraven & Baumeister, 2000) is unknown. Nonetheless, the energy mobilized by the cortisol response, combined with a physical task that was suited to recruiting these resources, may explain the collective results of the study.

### 4.1 Limitations and future directions

A limitation of this study was the absence of a control group, which will have provided clarity on whether controlled regulation increased performance and the cortisol response, or autonomous regulation reduced these consequences. Second, the study would have also benefitted from further details about the participants' physical fitness. Prior physical activity was included in the analysis as a covariate, which should have accounted for muscular endurance somewhat (i.e., physically active participants are more likely to possess greater muscular endurance). This point notwithstanding, wall sit performance has been shown to be sensitive to acute self‐control manipulations previously when not controlling for physical activity (e.g., Boat & Taylor, 2017). It is also worth noting that neither age nor gender emerged as a covariate of wall sit performance in this study. This tacitly supports the wall sit as a self‐control task, rather than one that is predicated on known individual differences in strength. In spite of these points, additional details about the participants' average weekly or monthly physical activities would further mitigate explanations related to physical fitness (e.g., muscular endurance) for performance on the wall sit. Third, the experiment was conducted by the lead researcher who was not blinded to the aims of the study. Every effort was made to ensure consistency between conditions, but this nonetheless increased the threat to internal validity. Fourth, we measured potential group differences in motivation at the end of the experiment in line with previous work (e.g., Moller et al., 2006). However, this failed to establish whether motivational effects never existed or had dissipated. Given that cortisol showed initial changes that subsided, it is more likely that motivational effects occurred but then dissipated, but this pattern cannot be confirmed. Related to this point, it would have been prudent to measure autonomous forms of extrinsic motivation (i.e., identified regulation; personal relevance to the participant; Ryan & Deci, 2019), rather than intrinsic motivation (i.e., fun and interest) to reflect the motivational manipulation more accurately.

Stress is a theoretically important yet poorly understood self‐control mechanism (Baumeister & Vohs, 2016). A key question raised by this study is whether the difference in cortisol levels between conditions influenced performance on the wall sit. This study was unable to discern whether the increased cortisol observed in the controlled condition may have aided wall sit performance (i.e., a physiological effect), or whether the difference in wall sit performance was independent of the observed changes in cortisol (i.e., a psychological effect). Future work should therefore test (a) if stress directly affects self‐control performance and depletion; (b) whether this effect varies across physical and cognitive tasks; and (c) the impact of more cognitively demanding self‐control tasks on stress and performance. These research suggestions will hopefully provide greater insight into the mechanisms underpinning self‐control.

### 4.2 Conclusion

The results of this study support a growing body of literature documenting the psychophysiological benefits of higher quality motivation (see: Steel et al., 2021). Participants exposed to a condition emphasizing autonomous regulation experienced an attenuated cortisol response in contrast to those in a controlled regulation condition who experienced an increase in cortisol. However, contrary to predictions, participants in the controlled regulation condition performed better on physical self‐control tasks when compared with the autonomous regulation condition. This implies that controlled regulation may have short‐term performance benefits in fairly simple physical tasks, but there is an associated psycho‐physiological cost for increased performance.

## CONFLICT OF INTEREST

We have no known conflict of interest to declare.

## AUTHOR CONTRIBUTIONS

**Richard Steel:** Conceptualization; Data curation; Formal analysis; Investigation; Methodology; Project administration; Visualization; Writing‐original draft. **Nicolette C. Bishop:** Conceptualization; Methodology; Supervision. **Ian Mark Taylor:** Conceptualization; Funding acquisition; Supervision; Writing‐review & editing.
